# CidA and LrgA: a “Hole” Lot More than Programmed Cell Death

**DOI:** 10.1128/mbio.00761-22

**Published:** 2022-05-24

**Authors:** Maisem Laabei, Seána Duggan

**Affiliations:** a Department of Biology and Biochemistry, University of Bath, Bath, United Kingdom; b Medical Research Council Centre for Medical Mycology, University of Exeter, Exeter, United Kingdom

**Keywords:** *Staphylococcus aureus*, programmed cell death, pyruvate

## Abstract

What do programmed cell death (PCD) and carbohydrate metabolism by-product transport have in common? Intriguingly, both processes involve the *cidABC* and *lrgAB* operons in the major human pathogen Staphylococcus aureus. Previously, CidA and LrgA have been studied in the context of programmed cell death, but a second function in overflow metabolism is increasingly evident. New work from J. L. Endres, S. S. Chaudhari, X. Zhang, J. Prahlad, et al. (mBio 13:e02827-21, 2022, https://doi.org/10.1128/mBio.02827-21) combining a lysis cassette, mutagenesis, and classic microbiology demonstrates that CidA and LrgA function as holins to support endolysin-induced lysis. But that’s not all—the *lrgAB* operon also facilitates pyruvate uptake during microaerobic and anaerobic growth. This commentary highlights the main findings from this work and places them in context of the literature to date. Finally, as these proteins are highly conserved and carry out disparate functions of great importance, it is tempting to speculate future work will elucidate the link between S. aureus lysis and pyruvate metabolism.

## COMMENTARY

Holins represent one of the largest nonorthologous functional homolog families. They span prokaryotes and eukaryotes and as such are of broad interest. Initially characterized as bacteriophage proteins, they have since gained prominence for roles in bacterial programmed cell death (PCD) due to their similarity to the small membrane-associated eukaryotic apoptotic Bcl-2 proteins. Holins are small membrane proteins required for host cell lysis by bacteriophages. Typically, they contain at least one, often four, transmembrane domains, are hydrophobic, and oligomerize in the cytoplasmic membrane. As a protein family, holins harbor vast variation in structure and mode of action. Essentially, they function by allowing a hole to form in the membrane, through which cytoplasmic murein hydrolase can escape and degrade peptidoglycan, diminishing membrane potential and resulting in cell death (1). Holins can be categorized as canonical holin endolysins resulting in pores between 35 and 340 nm and a pinhole SAR (signal-arrest-release) endolysin resulting in 2-nm pores. Given the system used to demonstrate holin activity, Endres et al. suggest CidA and LrgA to be members of the former canonical category (2).

In the present study, the authors build on their long-standing interest in the role of PCD and metabolism in biofilm development. Previous work demonstrated that gene products of the *cidABC* and *lrgAB* operons play a role in PCD regulation during biofilm development in response to carbohydrate metabolism, and these systems are controlled by the LysR-type transcriptional regulator, CidR (3). In their article, they hone in on the mechanism of key proteins of interest in the process of PCD in Staphylococcus aureus. To show holin-specific functionality, a lysis cassette in Escherichia coli was adapted to allow CidA and LrgA form pores in bacterial membranes. Next, His-tagged CidA and LrgA were incorporated into synthetic vesicles, where they localized to the membranes. The vesicles were loaded with dye and leakage determined. While both proteins resulted in dye leakage to the extravesicle space, CidA did so at a lower protein/lipid ratio than LrgA, indicating that CidA is the more efficient of the two holins. These experiments are important because they unequivocally demonstrate the holin-like function and bring a greater degree of certainty to the building picture of CidABC and LrgAB mechanisms than previous approaches have. LrgAB is a functional holin in its own right. Thus, it will be interesting to see how the previous work showing antiholin activity could be addressed in the future.

Next, *lrgAB* gene expression was shown to be induced in microaerobic growth, where it was required for pyruvate utilization. It is intriguing to consider the context of a protein with dual functions in lysis and metabolite import under specific conditions. These data pose the following questions. (i) What is the link between *cidABC-* and *lrgAB-*mediated transport of carbohydrate by-products of metabolism and PCD? (ii) How does carbon overflow potentiate cell death? The literature points to two mechanisms. First, the consumption of intracellular pyruvate by the pyruvate:menaquinone oxidoreductase CidC protein generates acetate, which can result in cytoplasmic acidification and thus generation of reactive oxygen species causing cell death. To combat this, generation of the neutral acetoin by α-acetolactate decarboxylase (AlsD) prevents the overacidification of the cytoplasm, and cells survive (4). Second, acetic acid has been shown to induce both *lrgAB* and *cidABC* operons and murein hydrolase activity (5), linking carbohydrate metabolism and cell lysis. Furthermore, the holin activities of CidA and LrgA are predicted to influence membrane depolarization and disruption of the proton motive force, resulting in an increase in localized pH triggering murein hydrolase activity and lysis. In their most recent work, Endres et al. (2) discuss *lrgAB* in the context of Bacillus subtilis and Streptococcus mutans *lrgAB* operons, where they function as intracellular pyruvate regulators, and one may hypothesize that LrgAB-mediated pyruvate shuttling feeds into the above systems, where excess carbon flow transported by *lrgAB* regulates cell death via modulation of intracellular acidification and dissipation of the cell membrane potential. The role of acetic acid as an inducer of cellular change analogous to eukaryotic apoptosis was previously seen in yeast species (6). Furthermore, acetate has been shown to activate apoptosis in cancer cell lines. This hints at the possibility *cid* and *lrg* systems might play similar roles linking carbohydrate metabolism to PCD in S. aureus (7). These points support the notion that within a biofilm, distinct physiological signals such as by-products of carbohydrate metabolism coordinate differential expression of systems involved in lysis and cell death, thus maintaining biofilm homeostasis. Recent work by Harper et al. linked pyruvate metabolism to an increase in the production of pore-forming leukotoxins (8). In response to pyruvate, S. aureus altered expression of *cidB* and *cidC*. However, *lrgAB* was not affected, presumably because those experiments were conducted under aerobic conditions. Therefore, pyruvate metabolism in S. aureus may contribute to lifestyle and virulence in ways we have not yet uncovered.

**FIG 1:**
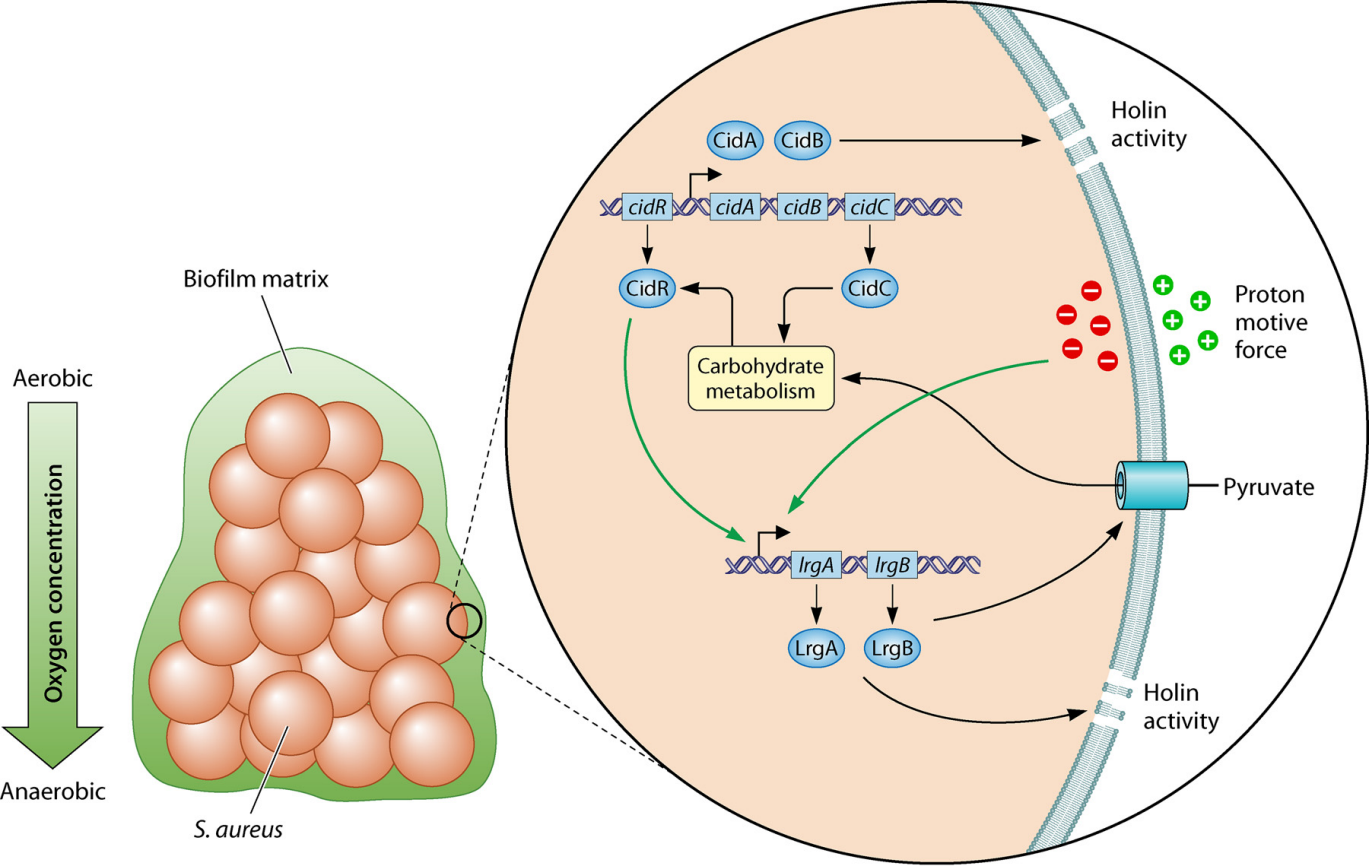
Schematic of the regulatory network of *cidABCR* and *lrgAB* operons and their roles in holin activity and pyruvate import during biofilm growth. CidAB and LrgAB function as holins under normal conditions, while LrgA and -B perform pyruvate transport function under anaerobic conditions. Membrane potential triggers transcription of the *lrgAB* operon with function in pyruvate import and holin formation. Pyruvate feeds into carbon metabolism and is a cue for CidR regulation of the CidABC operon. (Adapted from reference 9.)

The stratified nature of biofilms requires multiple strategies for bacteria to adapt. While some cells exist in an aerobic atmosphere, others deeper in the biofilm exist under microaerobic and even anaerobic conditions. Therefore, biofilm growth could be the relevant niche in which S. aureus tied PCD and overflow metabolism together (depicted in Fig. 1). Furthermore, it is an elegant design for CidA to function better as a holin, while LrgA is a less-efficient holin but has a dual function as a metabolite transporter. During biofilm growth, S. aureus cells can undergo cell death—as a result of a shutdown of metabolism or as a method of releasing extracellular DNA as an adherence molecule (9). Therefore, this niche would be suitable for S. aureus to combine the functions of PCD and overflow metabolism. While obviously technologically challenging, it would be interesting to see holin functionality investigated in biofilm and under subaerobic conditions in the future.

The authors touch on some open questions that are current lines of investigation in their lab. First, routine regrowth in the minibax control, but not CidA and LrgA expression constructs, is speculated to be due to plasmid curing or, more interestingly, a potential accumulation of mutations conferring resistance to minibax lysis. It will be interesting to address why this is not the case for CidA and LrgA. It is further tempting to speculate that LrgA is the lesser of two holins because it sacrifices holin functionality to transport pyruvate. Finally, the authors leave the reader thinking about the current investigations in the Bayles lab on LrgAB export of pyruvate. Hopefully, those experiments will go well, and the community will not have to wait too long for the next installment of the Cid-Lrg story.
